# MiR-6835 promoted LPS-induced inflammation of HUVECs associated with the interaction between TLR-4 and AdipoR1 in lipid rafts

**DOI:** 10.1371/journal.pone.0188604

**Published:** 2017-11-30

**Authors:** Jiao Liu, Guang Li, Chuang Chen, Dechang Chen, Qingshan Zhou

**Affiliations:** 1 Department of Critical Care Medicine, Renmin Hospital of Wuhan University, Wuhan, Hubei, People’s Republic of China; 2 Department of Breast and Thyroid Surgery, Renmin Hospital of Wuhan University, Wuhan, Hubei, People’s Republic of China; 3 Department of Emergency and Critical Care Medicine, Shanghai Changzheng Hospital, Second Military Medical University, Shanghai, People’s Republic of China; Universitat des Saarlandes, GERMANY

## Abstract

**Background:**

High mortality rate of critically-ill patients could be induced by sepsis and septic shock, which is the extremely life threatening. The purpose of this work is to identify and evaluate the potential regulatory mechanism of LPS-induced inflammation associated with miR-6835 and lipid rafts in HUVECs.

**Methods:**

The 3’ UTR luciferase activity of AdipoR1 was detected, which was predicted the potential target gene of miR-6835. Moreover, the treated HUVECs with or without inhibitors or mimics of miR-6835 were used. Furthermore, the bio-functions of HUVECs were explored. The protein expression levels of SIRT-1, AMPK, and AdipoR1 were assessed, which were involved in the AdipoR1 signaling pathway. Then, the interaction between TLR-4 and AdipoR1 in lipid rafts and its mediation role on LPS-induced inflammation was investigated in HUVECs.

**Results:**

MiR-6835 targeted directly on AdipoR1, and suppressed its expression in mRNA (mimics of miR-6835: 0.731±0.016 vs control: 1.527±0.015, *P*<0.001) and proteins levels, then regulated protein expression of SIRT-1 and AMPK, which were the downstream target genes of AdipoR1 signaling pathway. MiR-6835 enhanced LPS-induced inflammation process in HUVECs (TNF-α: LPS+mimics of miR-6835: 1638.51±78.43 vs LPS: 918.73±39.73, *P*<0.001; IL-6: LPS+mimics of miR-6835: 1249.35±69.51 vs LPS: 687.52±43.64, *P*<0.001), which was associated with the interaction between TLR-4 and AdipoR1 in lipid rafts.

**Conclusions:**

MiR-6835 is the key regulator of LPS-induced inflammation process in HUVECs. The interaction between TLR-4 and AdipoR1 mediated by lipid rafts at membrane of HUVECs with inflammation process induced by miR-6835. Our results demonstrated a hopeful strategy for treatment on sepsis by aiming at lipid rafts and miR-6835.

## Introduction

Sepsis and septic shock is an extremely life threatening, and can lead to high mortality rates in critically-ill patients. It is mostly associated with insufficiency between demand of cells and the delivery of oxygen, which caused the beginning of multi-organ dysfunction [1]. Sepsis is characterized by an overwhelming host response and development of remote organ failure [2]. Despite the development and clinical testing of numerous novel therapeutic compounds, sepsis/septic shock is still accompanied by high mortality rates reaching up to 50% of patients in the intensive care units worldwide [3, 4]. Through a wide variety of causes generalized activation of the endothelium, and then leads to shift to a pro-inflammatory phenotype. Once an inflammatory response has been instigated by endogenous mediators, such as cytokines, or components of the bacterial cell wall [5, 6], a large number of host-derived mediators are capable to further activate endothelial cells. The pro-inflammatory phenotype results in promotion of endothelial dysfunction, leukocyte trafficking and a pro-thrombotic state which potentially may lead to thrombotic micro angiopathy (TMA), remote organ dysfunction and death [7–9].

LPS (lipopolysaccharide) could activate TLR-4 (Toll-like receptor 4). LPS is a constituent of G^-^ bacteria (Gram-negative bacteria), which could cause manufacture of mediators related to pro-inflammatory process, and then target to eradicate bacteria. In host, the in normal regulation on inflammation response induced by LPS could lead to the systemic inflammatory process called sepsis. The TLR4 is typically activated at first, then binds to LPS and the protein of CD14, which located at the micro-domains of cell membrane named rafts constructed with expensive sphingolipid and cholesterol. Furthermore, LPS is transferred to the complex of MD2/TLR4 mediated by CD14, then induce production type I interferon and cytokines of pro-inflammatory [10]. The growing literatures hold up the crucial role of pro-inflammatory TLR4 signaling associated with raft integrity [11].

These studies point out the key function of microRNAs (miRNAs) on regulating expression at post-transcriptional level [12]. The miRNAs are non-coding RNA and tiny molecules, which supervise expression of gene through matching 3′ UTRs (un-translated regions) of target mRNA, then control its destabilization of gene or repression gene expression in translational level by incompletely pairing with its 3′ UTRs of mRNA. Furthermore, the aimed gene expression was down-regulated. The target genes were associated with ample process involved in cell biological functions, such as dysregulation of the different pathogenesis (including inflammatory disorders) [12]. The numerous of miRNAs maybe the marker of inflammation mediators, too [11–13].

Lipids and proteins are dynamically assembled in lipid rafts, then it is used as the signal transduction platform harbored many regulatory molecules and receptors [14]. It is found that changes of lipid rafts are commonly related to a great deal of human diseases. Moreover, through aiming at proteins in rafts using miRNAs, the domains of rafts could be perturbed [14]. The sphingolipid- and cholesterol-rich micro-domains are named lipid rafts, which are specialized plasma membrane [15]. The significance relationship between lipid rafts and LPS-induced inflammation has been elucidated in recent years [16–20].

In HUVECs, the biological effects induced by miR-6835 or its target genes were unclear. The main scope of our work was to identify the key microRNA, which could affect the inflammation process through regulating the genes expression associated with AdipoR1 signaling pathway. Moreover, whether the key microRNA regulating the inflammation process was related with TLR-4 or lipid rafts.

## Materials and methods

### Cell lines and culture

HUVECs was obtained from ATCC (American Type Culture Collection) (Manassas, VA). Cells were cultured with DMEM, 10% FBS (fetal bovine serum, and bi-antibiotics (Penicillin and Streptomycin; Sigma, USA) at 37°C in a 5% CO2 atmosphere. Four to six passage number of HUVECs when the cells reached 80–90% confluence was used in this experiment. During the logarithmic growth phase, cells were exposed to TNF (10 ng/mL). In the case of the existence of mycoplasma contaminated cell culture in quarantine and the absence of separate incubators, only flasks in a plastic box with lid should be used. Never use plates and unsealed dishes in quarantine. In the case of suspected cultures, handling them at the end of the workday after all other cell culture work is completed, using separated media and reagents, and finally disinfecting the laminar flow hood after working.

### Extract of RNA and miRNA clone

Total RNA was extracted from HUVECs with TRIzol (Sigma, USA) according to the protocol, and then isolated using mirVana RNA isolation kit to get rid of the smaller RNA (<200 nt) base on the manufacturer's instructions. The open code frame of miR-6835 was cloned into vector with DynaExpress miRNA Cloning Kit (BioDynamics Laboratory, Inc., USA). Total RNA was prepared as a mixture of RNAs isolated from HUVECs with 3 different donors and enriched twice by RNA precipitation. A total of 564.91 and 318.73 μg of RNA was acquired and separated on a denaturing polyacrylamide gel to isolate small RNAs of 18–28 nts in length. Purified 18–28-nt-long RNAs were dephosphorylated by alkaline phosphatase and subjected to phenol/chloroform extraction with ethanol precipitation. The 3′-linker was ligated to the dephosphorylated RNAs of 18–28 nts. The ligated products were blocked at the 3′-end to prevent circularization via 5′-linker ligation and purified on a polyacrylamide gel. The resulting miRNAs 36 to 46 nts in length were cut and extracted from the gel. The products were ligated with ribonucleotide 5′-linker at the 5′-end after their phosphorylation, followed by polyacrylamide gel electrophoresis purification as previously discussed. The resulting miRNAs with 2 linkers ranging from 53 to 63 nts were reverse-transcribed to make cDNA. After amplification, PCR products were analyzed on a 3% agarose gel and cloned into a T vector (Promega). The isolated putative clones were transformed into DH5α cells using a DokDo Mini-prep Kit (ELPIS-Biotech, Korea), sequenced, and analyzed (Invitrogen, USA).

### Reporter gene assays

The integrity cDNAs come from mRNAs of AdipoR1 3′UTR was predicted and obtained from HUVECs, and then cloned into luciferase reporter vector, pmirGLO (Dual-Luciferase® Reporter Assay System, Promga, USA), which could express luciferase of renilla and firefly. There was not target sequence of miRNA in control, the 3′UTRs of AdipoR1 cloned in reverse orientation was used as control [21]. Besides, the seed sequence of miR-6835 was complementary to the position of 3′UTR of AdipoR1, which region located at position 38–44, GUGCUUU. The mutated 3′UTR of AdipoR1 was constructed with Site-Mutation kit (Promega, Madison, WI, USA). The luciferase activity analysis in HUVECs was studied with analyzer VICTOR using Dual-Glo ® Luciferase Assay System (Promga, USA).

### Transfection of miR-6835 mimics

To investigate whether AdipoR1 was regulated by microRNA in HUVECs, online Targetscan (http://targetscan.org/), a software were firstly used to predict miRNAs which aimed at AdipoR1. Then we obtained many forecast results including miR-6835 (Table 1).

**Table 1:** Predicted consequential pairing of target region of AdipoR1 (top) and miRNA-6835 (bottom).

	Predicted consequential pairing of target region (top) and miRNA (bottom)	Site type	Context++ score	Context++ score percentile	Weighted context++ score	Conserved branch length	P_CT_
Position 38–44 of ADIPOR1 3' UTR	5' GGAGGAACUUCCCAAGUGCUUUU	7mer-m8	-0.29	99	-0.29	3.018	N/A
	|||||||						
hsa-miR-6835	3' GACCCUCUGUCUUUUCACGAAAA						

It revealed miR-6835 mimics: sense 5’-GGAGGAACUUCCCAAGUGCUUUU-3’ and antisense 5′-GACCCUCUGUCUUUUCACGAAAA-3′. NC (negative control was showed: sense 5′-ACGUGACACGUUCGGAGAAUU-3′ and antisense 5′-AAUUCUCCGAACGUGUCACGU-3′, which was not homologous with the genome sequences of human. Moreover, miR-6835 inhibitors were revealed: 5′-GUGGUCACCAUCUUCCCUU-3′. The miR-6835 mimics or its inhibitors was transfected with Lipofectamine 2000 (Invitrogen, USA) according to the manufacturer’s protocol. One day before transfection, seed 0.5–2×10^**5**^ cells per well in 500 μl of growth medium without antibiotics to attain 90–95% confluence at the time of transfection. For each transfection sample, prepare DNA-Lipofectamine 2000 complexes as follows: dilute 0.8 μg DNA in 50 μl Opti-MEM I (Invitrogen Catalog No. 31985, USA) without serum, and then mix gently. Mix the stock Lipofectamine 2000 gently before use, and then dilute 2 μl in 50 μl Opti-MEM I. Mix gently and let it stand for 5 min at room temperature. Five minutes after dilution of Lipofectamine 2000, combine the diluted DNA with the diluted lipid (total volume is 100 μl). Mix gently and let it stand for 20 min at room temperature to allow the DNA–Lipofectamine 2000 lipoplexes to form. The solution may appear cloudy, but this will not inhibit transfection. DNA–Lipofectamine 2000 complexes are stable for 6 h at room temperature. Add 100 μl of transfection complex to each well containing cells and medium. Mix gently by rocking the plate back and forth. Incubate the cells at 37°C in a humidified incubator with 5% CO_**2**_ for 24–48 h until they are ready to assay. The concentration effects induced by miR-6835 were identified using qRT-PCR method.

### qRT-PCR assay

The assays of TaqMan miRNA were used to identify the miR-6835 expression in HUVECs. SYBR Green kit was used in qRT-PCR analysis, the assays of qRT-PCR were conducted with Real-Time PCR Detection System (Bio-Rad, Berkeley, CA, USA). The special primer sequences of AdipoR1 was revealed: upstream 5′-CAGATTTTCCATGTCCTGGTG-3′ and downstream 5′-CGGAATTCCTGAAGGTTGG-3′ and glyceraldehyde-3-phosphate dehydrogenase **(**GAPDH) was as control: forward 5′-ctcatgaccacagtccatgcc-3′ and downward 5′-ggcatggactgtggtcatgag-3′.

Normalize input RNA samples to a concentration of 1 ng/μL. Thaw RT kit (Invitrogen, USA) components on ice. Mix gently and centrifuge briefly. Calculate number of RT reactions, including no-input control. Make RT master mix on ice, using attached spreadsheet to scale the following per reaction mixture: 0.15 μL 100mM dNTPs (with dTTP), 1 μL MultiScribe^**TM**^ Reverse Transcriptase (50U/μL), 1.5 μL 10×Reverse Transcription Buffer, 0.19 μL RNase Inhibitor (50U/μL), 8.16 μL Nuclease-free water, Total volume 11.00μL. Mix and certrifuge briefly. Combine in 0.2 ml qPCR well: 1μL input RNA, 11μL RT master mix, 3 μL 5×RT primer. Seal, mix, and centrifuge briefly. Incubate on ice for 5 mins. Place in thermocycler and run the following program: 16°C 30 min, 42°C 30 min, 85°C 5 min, 4°C Hold. The reactions may be stored at -20°C at this point. Thaw frozen PCR kit (Invitrogen, USA) components on ice. Mix and centrifuge briefly. Swirl mastermix bottle gently to mix. Calculate number of samples, including no-input and no-template controls for each miRNA assay. Set up triplicate PCR reactions for each template/assay combination, using the attached spreadsheet to scale the following per reaction mixture: 0.5 μL TaqMan® Small RNA Assay (20×), 0.66 μL Product from RT reaction, 5 μL TaqMan® Universal PCR Master Mix Ⅱ(20×), no UNG, 3.84 μL Nuclease-free water, Total volume 10.00 μL. Mix and centrifuge briefly. Distribute into 0.2 ml qPCR wells, 10 μl per well. Seal, mix, and centrifuge briefly. Place in qPCR machine and run the following program: 1 cycle of: 95°C 10 min, 40 cycles of: 95°C 15 s, 60°C 60 s.

### Western blotting

Cells were collected and total proteins were extracted in 40 mM Tris-HCl (pH 7.4) containing 150 mM NaCl and 1% (v/v) Triton X-100 (Sigma, USA), supplemented with protease inhibitors. Protein concentration was determined using the bicinchoninic acid protein assay (Pierce, Rockford, IL, USA). Equal amounts of protein were resolved on 10% SDS-PAGE gels (Sigma, USA), and then transferred to a PVDF membrane (Millipore, Bedford, MA, USA). After blocking with 5% skimmed milk in TBST (Sigma, USA), then probed with antibodies against AdipoR1, SIRT-1, AMPK (1: 1000) (Cell Signaling Technology, Danvers, MA, USA), p-AMPK, TLR-4 (1: 1000) (Santa Cruz, CA, USA), β-actin (1: 2000) (Cell Signaling Technology, Danvers, MA, USA), respectively, at 4°C, overnight. After three times washes, blots were then incubated with horseradish peroxidase (HRP) conjugated secondary antibodies (Cell Signaling Technology, Inc., Boston, MA, USA). Immunoreactive bands were visualized using the enhanced chemiluminescence (ECL) detection system (Promega, USA). The protein levels of the stripes were normalized based on the gray value of β-actin.

### Clonogenicity assays

Over- or down-expression of miR-6835 were induced for assessing its molecular biological functions in HUVECs with several experiments. The clonogenicity experiment was performed, cells colonies were fixed using formalin and then stained by crystal violet (Sigma, USA). Furthermore, the number of obvious colonies were figured out.

### Cell proliferation assay

Cell Count Kit-8 assay (CCK-8, Dojindo, Japan) was used as a qualitative marker for cell proliferation ability. After 48 h transfection with miR-6835 mimic, NC, HUVECs were seeded in 96 well plates in triplicate with 5×10^3^ cells per well. At 24 h, 10 uL of CCK-8 solution mixed with 90 uL of DMEM was added to each well. And after 2 h of incubation, the absorbance was measured at 450 nm.

### Analysis of cell migration

The migration ability of HUVECs were measured with wound scratch experiment. At first, HUVECs were scratched, then its movement was detected after transfection for 48 h. Then, the migration HUVECs were counted.

### Co-immunoprecipitation

After lysed, HUVECs was dealt with PMSF (Phenylmethanesulfonyl fluoride) (Sigma, USA), and Protein G Plus/Protein A Agarose Suspension (Santa Cruz, CA, USA) was used. Then, after using antibody for TLR-4 or AdipoR1, Agarose Suspension of Protein G Plus/Protein A was added again, respectively. Moreover, immunoprecipitates were performed with SDS-PAGE electrophoresis, respectively, then immunoblotting experiment were conducted with anti-AdipoR1 or anti-TLR-4, respectively.

### Confocal laser scanning

The TLR-4 and AdipoR1 cDNA sequence were respectively cloned into pEGFP-C1 or pDS-RED1-N1 vector. Then the transfection of expression vectors pDS-RED1-N1-AdipoR1 and pEGFP-C1-TLR-4 were performed in HUVECs. After 48 h of transfection, HUVECs were fixed and stained with DAPI (4', 6-diamidino-2-phenylindole) (Sigma, USA). In the end, HUVECs were observed and photograph were obtained with LSM 510 microscope (Zeiss, Germen).

### Purification of membrane and lipid raft protein

At first, the proteins of membrane were extracted from HUVECs [22]. Then the fractions of lipid rafts were purified from with density gradient ultracentrifugation using modified sucrose (Sigma, USA) [23, 24]. Finally, these lipid rafts were fractionated into 12 subfractions [25].

### Enzyme-linked immunosorbent (ELISA) experiment

The enzyme-linked immunosorbent ELISA assay kits (for IL-6 and TNF-α) were obtained from Biolegend (CA, USA). The levels of these cytokines were measured using these sandwich ELISA kits according to the manufacturer’s protocol.

### Statistical analyses

SPSS17.0 software was used to analyze the data. Values are expressed as mean±SD of experiments performed in triplicate. Data were analyzed by one-way ANOVA and Student’s *t*-test. Statistical significance was defined as p<0.05.

## Results

### miR-6835 suppressed AdipoR1 expression in HUVECs

**Fig 1:**
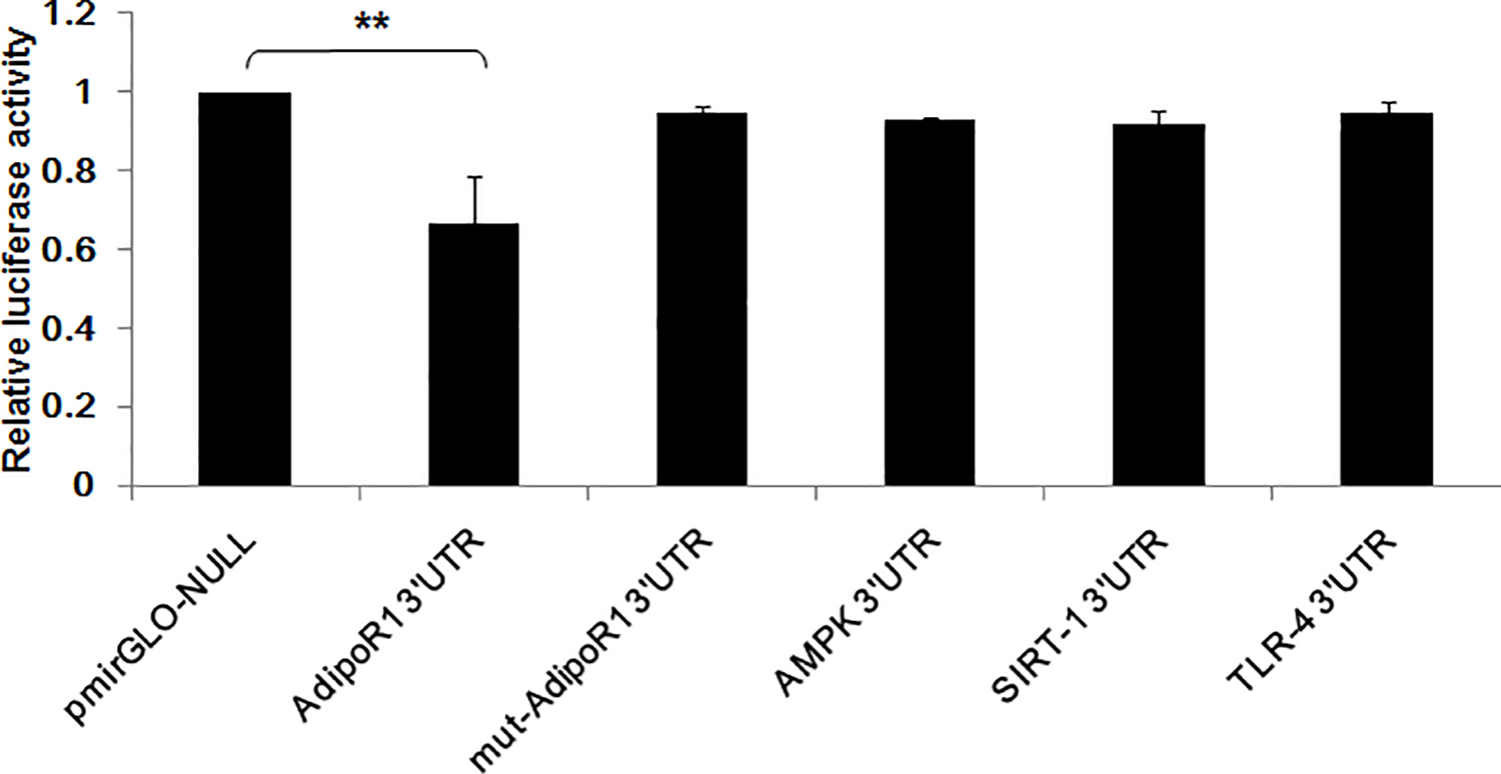
MiR-6835 targeted at AdipoR1 in HUVECs. The results showed the obviously down-regulated 3’ UTR activities of AdipoR1 in HUVECs, but no alternations were found while AdipoR1 with mutation. Moreover, miR-6835 could not directly target at AMPK, SIRT-1, and TLR-4, respectively. The data are presented as means±SD from three independent experiments. *P<0.05, **P<0.01.

To investigate whether AdipoR1 was regulated by predicted miR-6835 in HUVECs, hence, the identified assays were performed with luciferase activity using reporter gene assay. Our results indicated that in HUVECs, the relative down-regulated activities of 3’ UTR of AdipoR1 obviously were revealed (Fig 1) (mimics of AdipoR1 0.731±0.016 vs control: 1.527±0.015, *P*<0.001), but no alternations were found while AdipoR1 with mutation (Fig 1). This proved that AdipoR1 3’ UTRs was targeted by miR-6835, which could straight bond with AdipoR1 3’ UTRs. But, miR-6835 cannot directly bind with AMPK, SIRT-1, and TLR-4 3’ UTRs, respectively (Fig 1).

**Fig 2:**
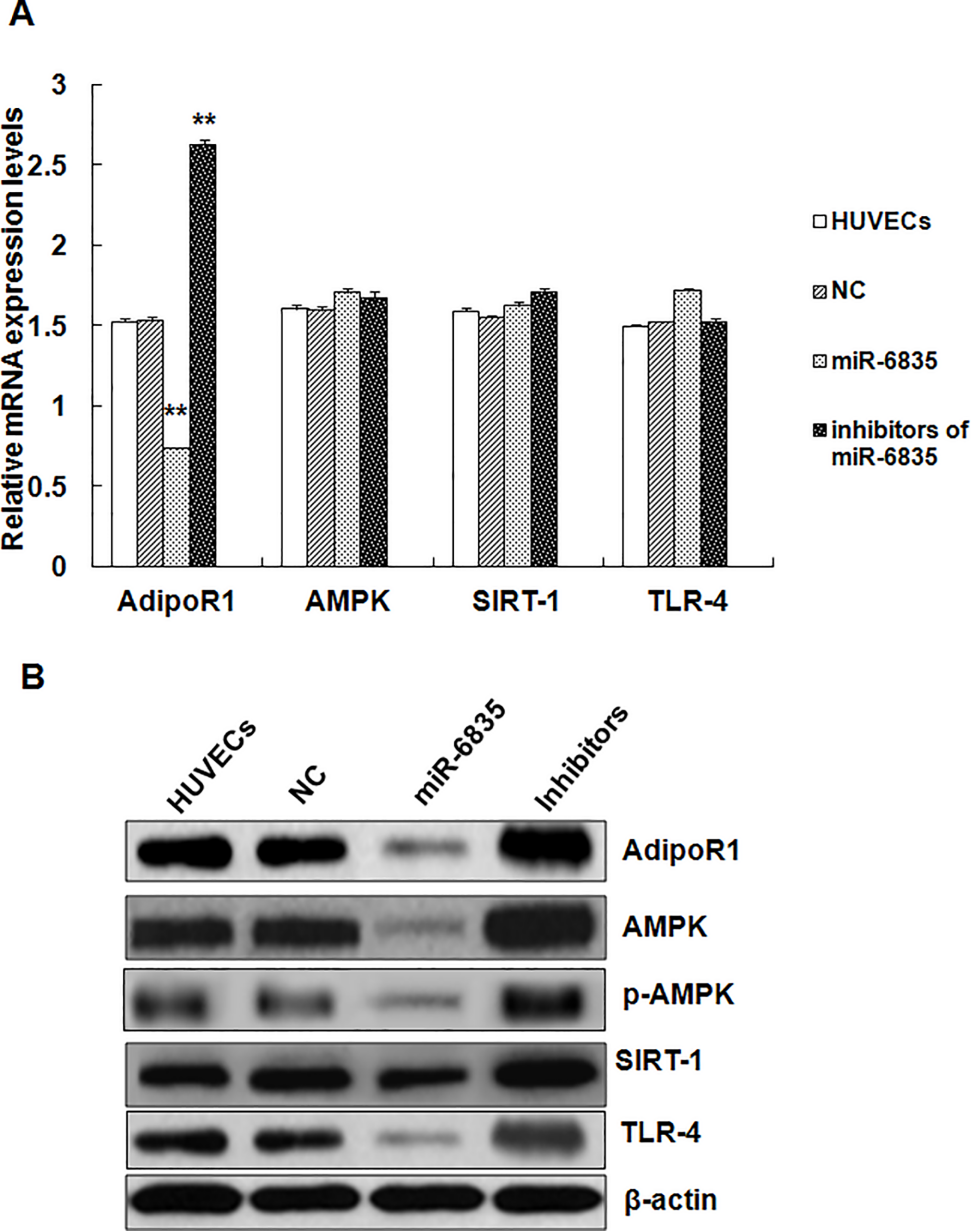
MiR-6835 suppressed genes expression of AdipoR1 pathway in HUVECs. Our results suggested that the mRNA and protein expression of AdipoR1 was suppressed by miR-6835, respectively (A and B). Moreover, mRNA expressions of AMPK, SIRT-1, and TLR-4 could not be influenced by miR-6835, but their proteins level (B). Additionally, the mRNA expression level of AdipoR1 could be promoted by inhibitors of miR-6835, however, which could not affect mRNAs expression level of AMPK, SIRT-1, and TLR-4 (A). The data are presented as means±SD from three independent experiments. *P<0.05, **P<0.01.

To evaluated the effects induced by miR-6835 on mRNAs or proteins expression of these genes in HUVECs, western blots and RT-PCR were conducted (Fig 2). These results suggested that the mRNA (mimics of miR-6835: 0.731±0.016 vs control: 1.527±0.015, *P*<0.001) and protein expression levels of AdipoR1 could be suppressed by miR-6835 (Fig 2). Furthermore, miR-6835 could not influence AMPK, SIRT-1, and TLR-4 expressions in mRNA level, in contrast, affected their proteins level (Fig 2B). Additionally, AdipoR1 expression in mRNA level (inhibitors of miR-6835: 2.618±0.038 vs control: 1.527±0.015, *P*<0.001) could be promoted by miR-6835 inhibitors, but which could not affect the mRNAs expression levels of AMPK, SIRT-1, and TLR-4 (Fig 2A). In conclusion, it indicated AdipoR1 expression was regulated directly by miR-6835 in mRNA and protein levels through bonding with 3′-UTR of AdipoR1, moreover, only influence protein expression level of these genes located its down-stream.

### Clonogenicity and growth of HUVECs was inhibited by miR-6835

**Fig 3:**
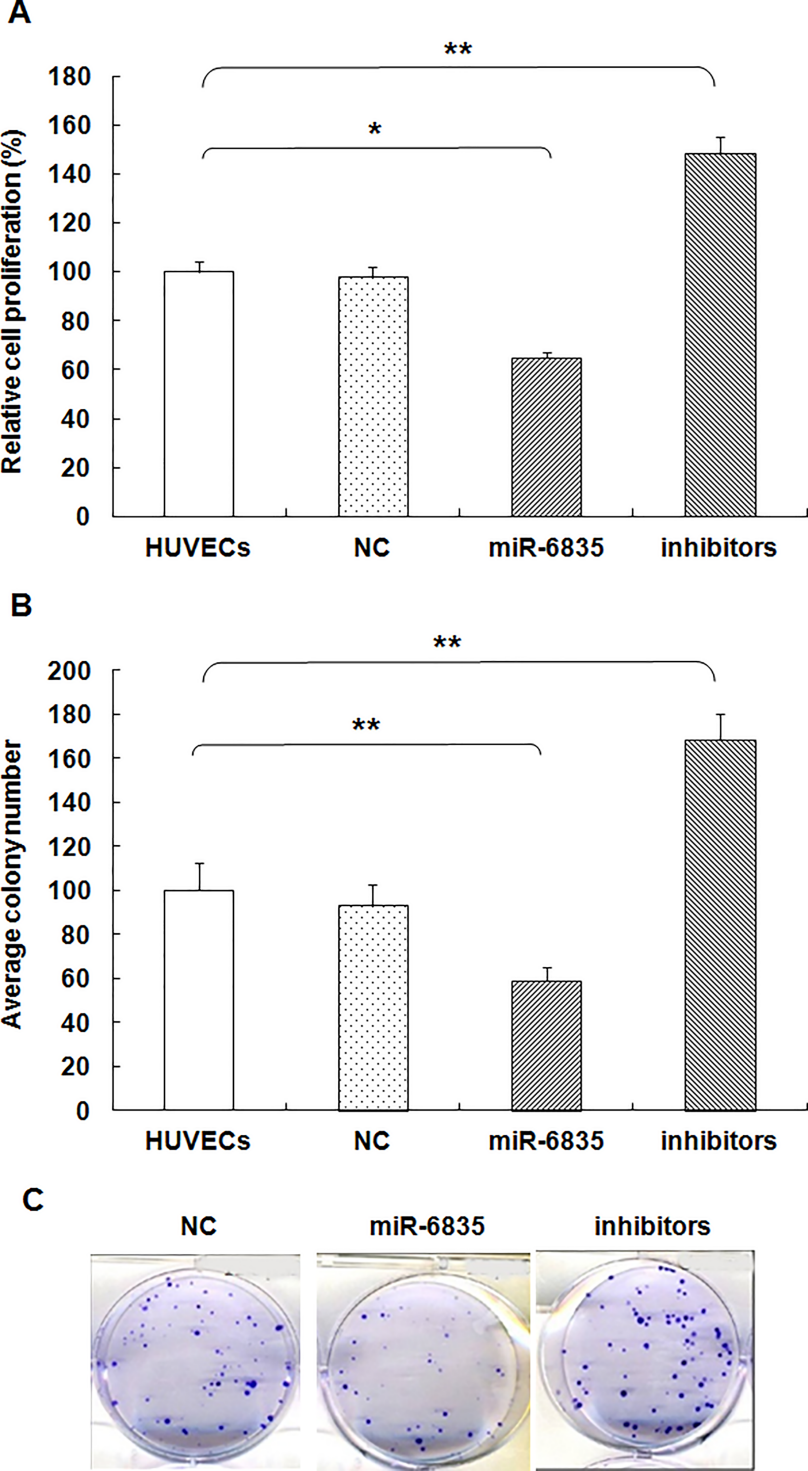
MiR-6835 inhibited clonogenicity and growth of HUVECs. (A) The proliferation of HUVECs was restrained by miR-6835 compared to control group. Furthermore, the inhibitors of miR-6835 promoted proliferation of HUVECs. (B and C) The clonogenicity of HUVECs was restrained by miR-6835 compared to control group. Furthermore, the inhibitors of miR-6835 promoted clonogenicity of HUVECs. The data are presented as means±SD from three independent experiments. *P<0.05, **P<0.01.

Compared to control group, HUVECs proliferation was suppressed by miR-6835 (mimics of miR-6835: 64.73±2.47 vs control: 100.00±4.18, *P*<0.001) (Fig 3A). But, miR-6835 inhibitors could promote the proliferation process in HUVECs (inhibitors of miR-6835: 148.66±6.73 vs control: 100.00±4.18, *P*<0.001) (Fig 3A). It suggested that miR-6835 inhibited HUVECs proliferation. Moreover, miR-6835 suppressed significantly the ability of colony formation in HUVECs (mimics of miR-6835: 58.73±6.66 vs control: 100.00±12.34, *P*<0.001). In contrast, miR-6835 inhibitors obviously promoted the ability of HUVECs (inhibitors of miR-6835: 168.64±11.38 vs control: 100.00±12.34) (Fig 3B and 3C, *P*<0.001). It demonstrated miR-6835 could restrain colony formation ability of HUVECs, too.

### MiR-6835 restrained migration of HUVECs

**Fig 4:**
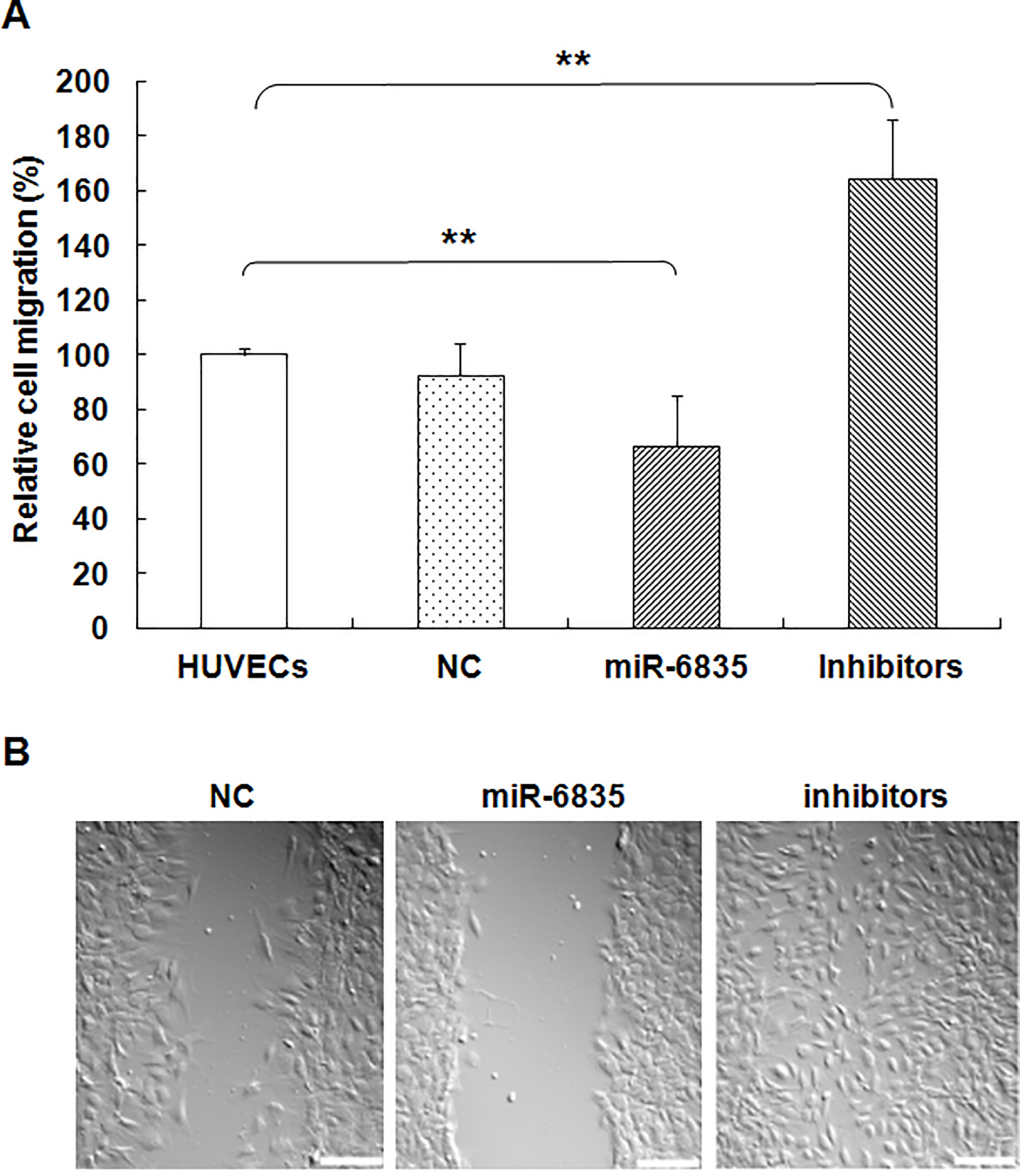
MiR-6835 restrained migration of HUVECs. I*n vitro*, the migration ability of HUVECs was significantly suppressed by mimics of miR-6835. On the contrary, the migration ability of HUVECS was enhanced by miR-6835 inhibitors. The data are presented as means±SD from three independent experiments. *P<0.05, **P<0.01. The scale bar of (B) was 400×.

Our results indicated that the migration ability of HUVECs *in vitro* could be restrained significantly by over-expression of miR-6835 (mimics of miR-6835: 64.91±8.43 vs control: 100.00±11.67) (Fig 4, *P*<0.001). In contrast, the ability of migration was enhanced by the inhibitors of miR-6835 in HUVECs (mimics of miR-6835: 144.36±18.31 vs control: 100.00±11.67, *P*<0.001) (Fig 4) From these data, it suggested that miR-6835 could restrain migration ability in HUVECs.

### AdipoR1 bonded with TLR-4

**Fig 5:**
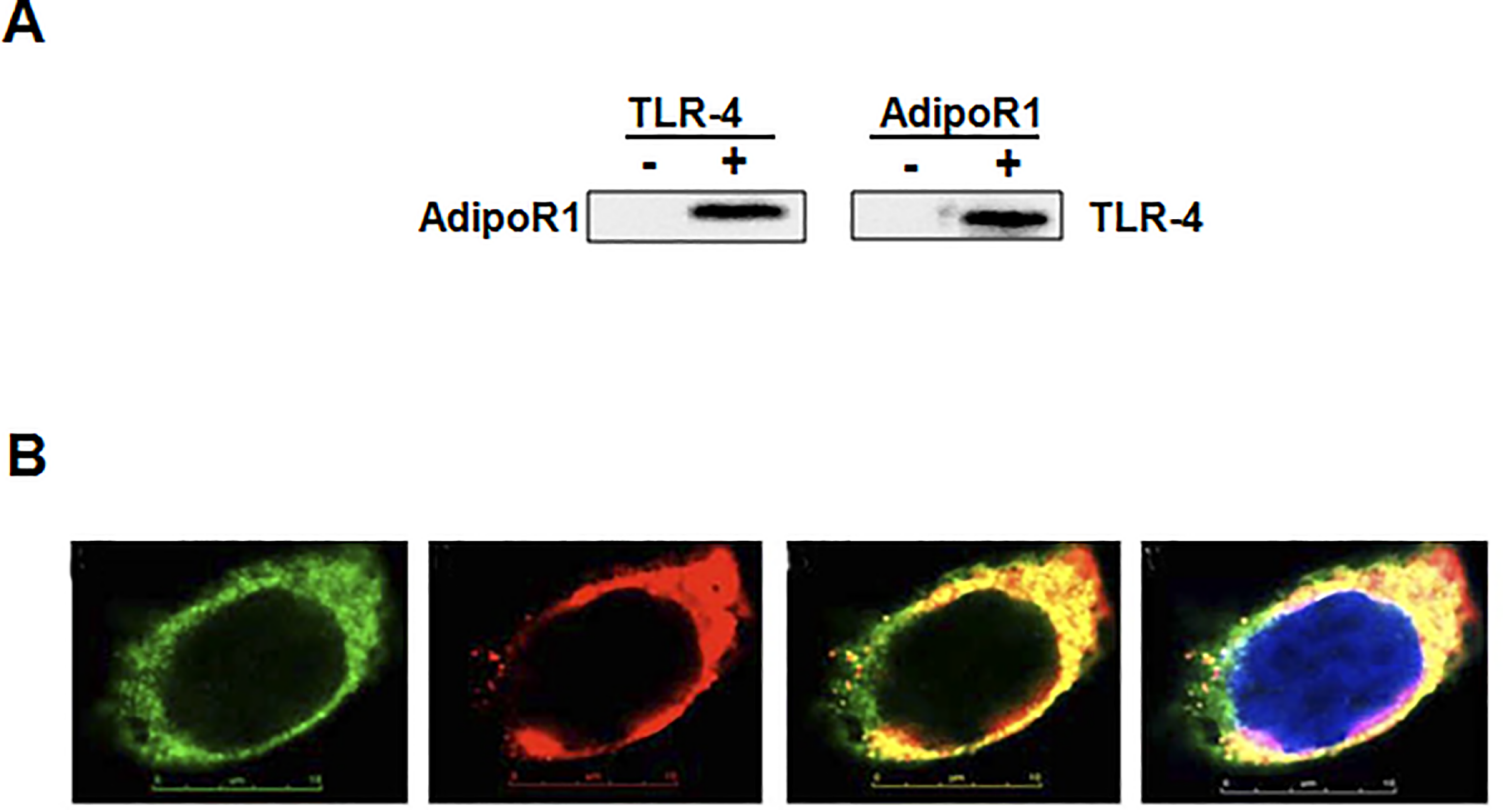
AdipoR1 could bond with TLR-4. (A) The results CO-IP’d (co-immunoprecipitated) assay was perfomed, and identified the interaction between AdipoR1 and TLR-4. (B) The confocal images demonstrated that both the two recombinant proteins of AdipoR1 and TLR-4 localized at cell membrane of HUVECs with overlaid exhibition.

The CO-IP experiment and technology of CLSM (confocal laser scanning microscopy) were used to analyze the interaction between AdipoR1 and TLR-4. As described above in part of methods, CO-IP’d (co-immunoprecipitated) assay was conducted, these results identified the interaction between AdipoR1 and TLR-4 (Fig 5A). AdipoR1 is a protein located at cell membrane of HUVECs. The images come from confocal scanning indicated the recombinant protein of TLR-4 and AdipoR1 produced by pEGFP-C1-TLR-4 (green) and pDS-RED1-N1-AdipoR1 (red) after transfection for 48 h, respectively. Moreover, both the two proteins localized at cell membrane with overlaid exhibition (Fig 5B). The overlaid image indicates that AdipoR1 overlapped with TLR-4 at the cell membrane. Our results identified the interaction between AdipoR1 and TLR-4 at the membrane of HUVECs.

### The inhibitors of miR-6835 induced migration of AdipoR1 into lipid rafts

**Fig 6:**
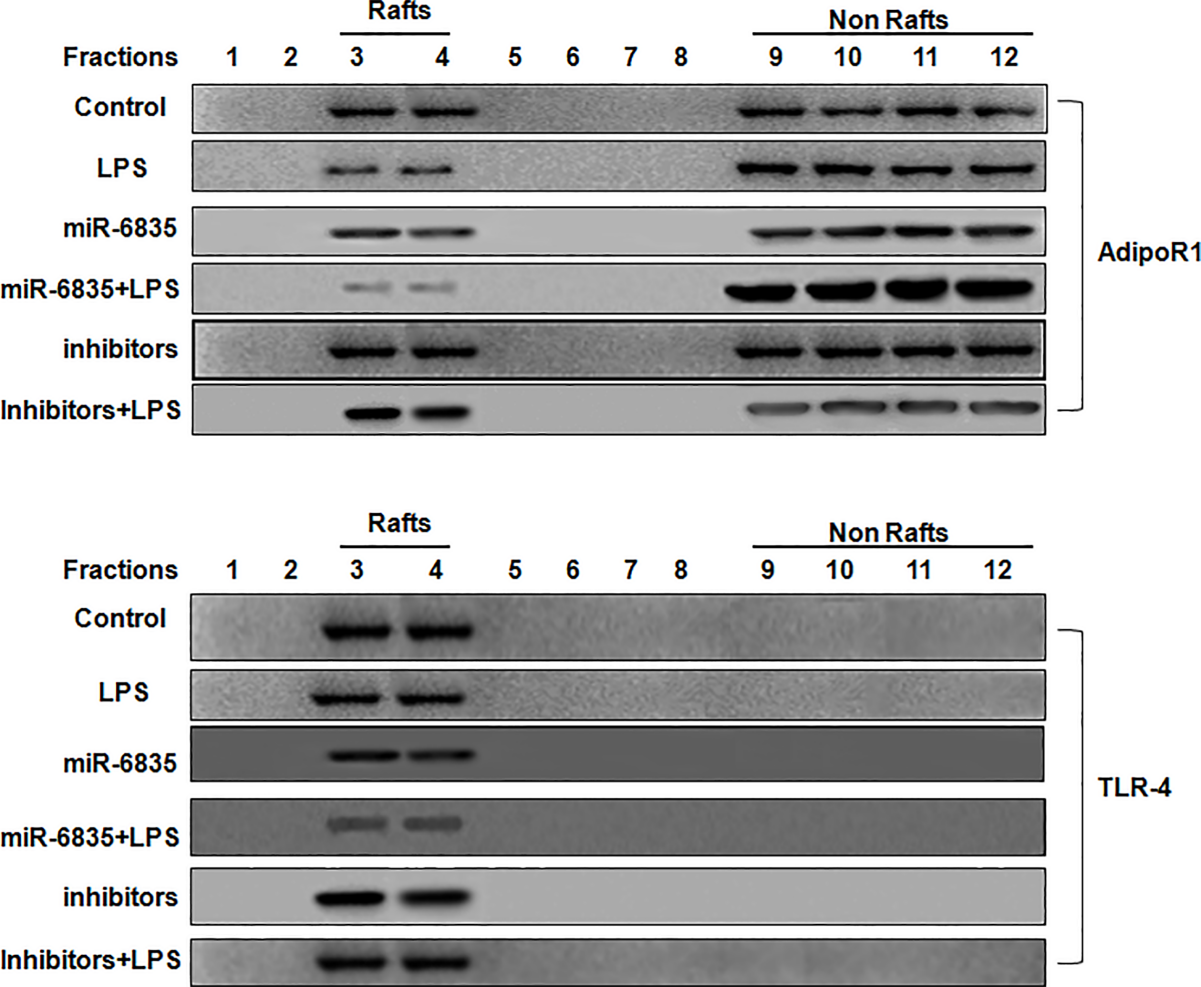
The inhibitors of miR-6835 induced migration of AdipoR1 into lipid rafts. Our results demonstrated that miR-6835 inhibitors of suppressed AdipoR1migration into lipid rafts, and then cut down the localization of AdipoR1 and TLR-4 in fractions of rafts. However, mimics of miR-6835 promoted this effect.

In recent, ample data demonstrated assembly platforms for functional receptor were provided by lipid rafts. AdipoR1 played a crucial factor in inflammation process of HUVECs induced by LPS, and maybe the potential therapy for patients with sepsis. The movability of AdipoR1 into lipid rafts is important to anti-inflammation process. Furthermore, the potential role induced by miR-6835 inhibitors was identified, it demonstrated the inhibitors of miR-6835 restrained migration of AdipoR1 into lipid rafts, and then cut down the localization of AdipoR1 in fractions of rafts as well as TLR-4. But, this effect was promoted by mimics of miR-6835 (Fig 6).

### MiR-6835 promoted LPS-induced inflammation response in HUVECs

As described above, we found that miR-6835 inhibit expression of AdipoR1 in HUVECs. To explore the role of miR-6835 on the inflammatory response in HUVECs, we investigated HUVECs stimulated with LPS. In this work, the cultured HUVECs was applied as a suitable model as in many studies. LPS is widely used to investigate the inflammatory response in both primary cell cultures and immortalized cell lines *in vitro*, or animal models *in vivo*. Consequently, LPS-induced endothelial inflammatory response was assessed by analyzing IL-6 and TNF-α expression.

**Fig 7:**
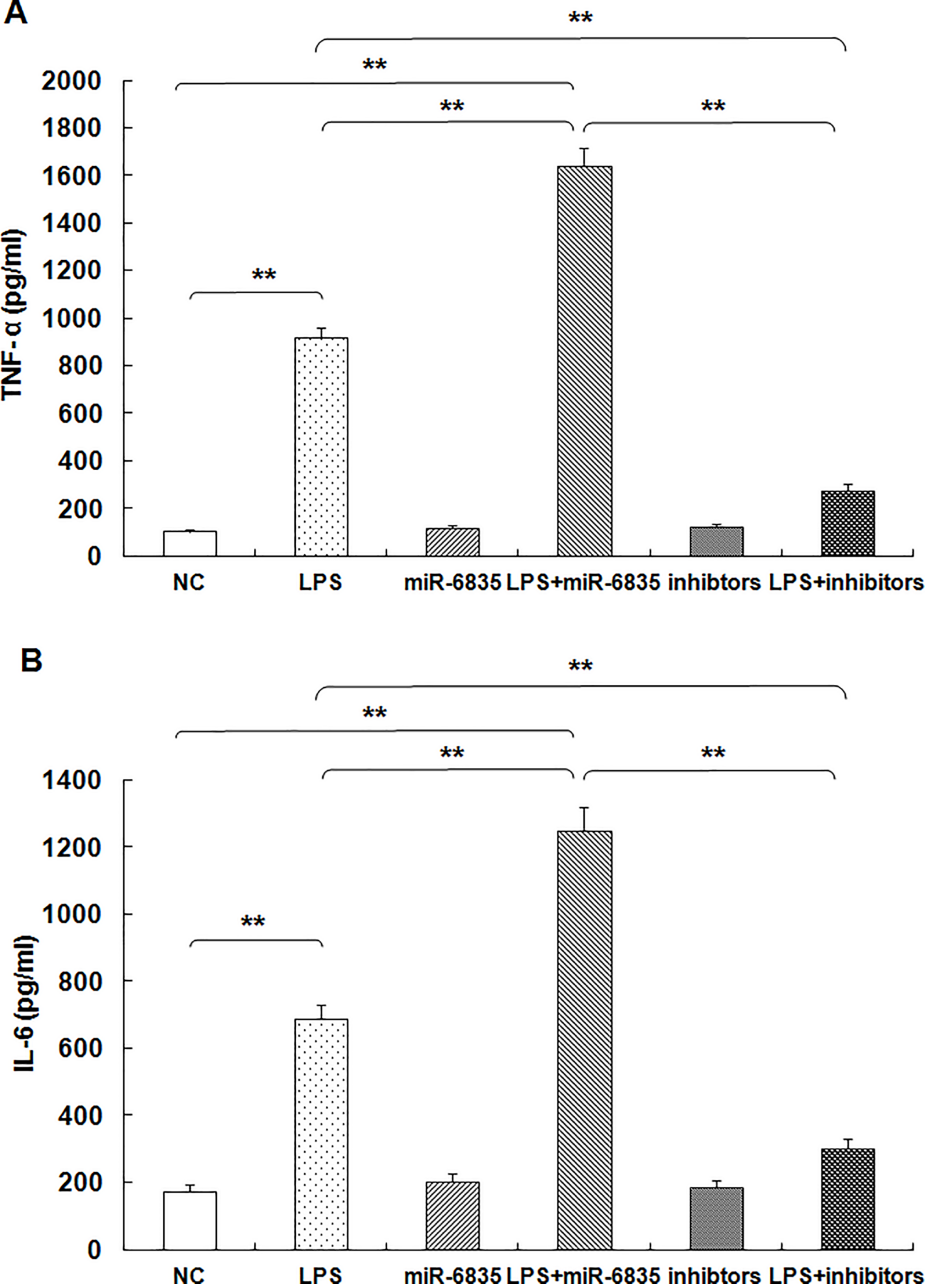
MiR-6835 promoted LPS-induced inflammation response in HUVECs. LPS induced high baseline expression levels of IL-6 and TNF-αin HUVECs. Moreover, after treating with above concentrations of LPS for 16 hours, HUVECs were transfected with 0.2 nM miR-6835 or its inhibitors. The results showed that miR-6835 promoted LPS-induced expression of IL-6 and TNF-α in HUVECs. But, the inhibitors of miR-6835 caused converse effect. The data are presented as means ± SD from three independent experiments. *P<0.05, **P<0.01.

These results of ELISA analysis indicated that LPS caused high baseline IL-6 and TNF-α expression levels in HUVECs. As expected, followed with the treatment of LPS, the expression level of TNF-α (LPS: 918.73±39.73 vs control: 108.45±9.26, *P*<0.001) and IL-6 (LPS: 687.52±43.64 vs control: 173.29±21.48, *P*<0.001) drastically increased almost 10-fold, respectively (Fig 7). The results of ELISA analysis showed that a 16 h exposure to LPS in HUVECs, the TNF-α and IL-6 level in supernatant of HUVECs increased obviously compared to control cells (Fig 7).

Moreover, after treating with above concentrations of LPS for 16 h, HUVECs were followed by transfection with 0.2 nM miR-6835 or its inhibitors for an additional culture of 48 h. The consequences indicated that miR-6835 could promoted LPS-induced IL-6 (LPS+mimics of miR-6835: 1249.35±69.51 vs LPS: 687.52±43.64, *P*<0.001) and TNF-α (LPS+mimics of miR-6835: 1638.51±78.43 vs LPS: 918.73±39.73, *P*<0.001) expression in HUVECs. It is noteworthy that the inhibitors of miR-6835 correspondingly caused the converse activities (Fig 7). It demonstrated that miR-6835 could significantly promote the inflammation process induced by LPS in HUVECs.

## Discussion

Sepsis and septic shock is the extremely life threatening [26]. The persistent dysfunction of multi-organ could be quickly induced by sepsis, which is acute systemic inflammatory response. It could be changed to hypo/late phase of inflammation in sepsis shifted from the hyper/early phase of sepsis [27, 28]. Ample data demonstrated that at hypo/late phase of inflammation in sepsis, host cannot effectively remove pre-existing or superadded infections [29–31]. The different treatment therapies were used to sepsis, and about more than 30 methods. It all targeted early phase of sepsis, but could not improve survival [32], thus most patients with sepsis died during late phase of sepsis. Many evidences supported many novel modalities for treatment on sepsis through suppressing immune response [29, 33].

In this work, we explored the mechanism related to LPS-induced inflammation in HUVECs, moreover, the gene expression control was investigated at transcriptional level. Though a few of molecular mechanisms in sepsis was well known, but how miRNA modulate the LPS-induced inflammation process mediated by lipid rafts in HUVECs was still indecipherable. Many elements including miRNAs perform important effect on regulation of gene expression, which was associated with LPS-induced inflammation process [34]. During dysregulation status of a great deal of genes, their epigenetic reprogramming was highly fatal by target at acute systemic inflammation process in sepsis [35]. MicroRNAs are associated with physiological and pathological processes such as inflammation. Adipose tissue could secreted adiponectin, which is an adipokine. It could handle biological roles by its receptors: Adiponectin receptor 1 (AdipoR1) and Adiponectin receptor 2 (AdipoR2). AdipoR1 could regulate the expression of AMPK (5′-adenosine monophosphate-activated protein kinase) [36]. But the microRNAs-mediated activity and expression of AdipoR1 during development process of inflammation is quite unclear.

In our study, online Targetscan (http://targetscan.org/), a software were used to predict miRNAs which aimed at AdipoR1. Therefore, we obtained many forecast results including miR-6835, and then the role of miR-6835 was searched after in development inflammation process. Further results of this work firstly demonstrated the obviously down-regulated 3’ UTR activities of AdipoR1 in HUVECs. This proved that AdipoR1 3’ UTRs was targeted by miR-6835, but, miR-6835 could not directly bind with 3’ UTRs of AdipoR1 pathway gene, including AMPK, SIRT-1, and TLR-4 [37]. We found that the mRNA and protein expression of AdipoR1 could be suppressed by miR-6835. But, miR-6835 could not influence the mRNA expressions level of AMPK, SIRT-1, and TLR-4, on the contrary, affected their proteins expression. Our results firstly confirmed AdipoR1 mRNAs are straight goals of miR-6835. In a word, it indicated AdipoR1 expression was regulated directly by miR-6835 in mRNA and protein levels through bonding with 3′-UTR of AdipoR1.

Vascular endothelial injury and hyperpermeability play an important role in the development of sepsis-induced organ dysfunction [38]. The present study was performed to investigate the role of miR-6835 in endothelial barrier dysfunction during sepsis. Then, over- or down-expression of miR-6835 were induced for assessing its molecular biological functions in HUVECs with several experiments. Compared to control group, the ability of proliferation, colony formation, migration, invasion of HUVECs was suppressed by miR-6835. But, miR-6835 inhibitors could promote these cellular biological process in HUVECs. Therefore, the inhibitors of miR-6835 may play the protective role in vascular endothelial barrier dysfunction by preserving the endothelial permeability during sepsis.

Badshah et al had found the colocalization of TLR4 and AdipoR1 receptors in BV2 microglial cells, which suggests that osmotin binds to AdipoR1 and inhibits downstream TLR4 signaling [39]. In this work, the CO-IP experiment and technology of CLSM (confocal laser scanning microscopy) were used to analyze the interaction between AdipoR1 and TLR-4 in HUVECs. Our results identified the interaction between AdipoR1 and TLR-4 at the membrane of HUVECs. Lipids and proteins are dynamically assembled in lipid rafts, then it is used as the signal transduction platform harbored many regulatory molecules and receptors. It is found that changes of lipid rafts are commonly related to a great deal of human diseases [40]. Moreover, through aiming at proteins in rafts using miRNAs, the domains of rafts could be perturbed. The sphingolipid- and cholesterol-rich micro-domains are named lipid rafts, which are specialized plasma membrane. The significance relationship between lipid rafts and LPS-induced inflammation has been elucidated in recent years [41, 42]. In recent, ample data demonstrated assembly platforms for functional receptor were provided by lipid rafts. AdipoR1 played a crucial factor in inflammation process of HUVECs induced by LPS [6, 37], and maybe the potential therapy for patients with sepsis. We found that the movability into lipid rafts of AdipoR1 is important to anti-inflammation process. Moreover, the potential anti-inflmmation role induced by miR-6835 inhibitors was identified, which restrained AdipoR1 migration into lipid rafts, and then cut down the localization of AdipoR1 and TLR-4 in fractions of rafts. It is well known that AdipoR1 play the crucial effect on the process of inflammation [37, 43], and maybe alternatively used as the possible therapeutic tactic for treatment on sepsis. The maneuverability of AdipoR1 in lipid rafts played important role on anti-inflammation process.

In conclusion, miR-6835 targeted directly on AdipoR1, and suppressed its expression in mRNA and proteins levels, then regulated expression of SIRT-1 and AMPK associated with its downstream signaling pathway. The growing literatures indicated miRNAs acted as key regulator microRNA to promote or inhibit inflammation process in HUVECs, and may contribute to sepsis diagnosis and treatment. We found that miR-6835 enhanced LPS-induced inflammation process in HUVECs, which was associated with lipid rafts by regulating TLR-4’s binding ability towards AdipoR1. MiR-6835 is the key regulator of LPS-induced inflammation process in HUVECs. The interaction between TLR-4 and AdipoR1 mediated by lipid rafts at membrane of HUVECs with inflammation effect induced by miR-6835. Our results demonstrated an hopeful strategy for treatment on sepsis by aiming at lipid rafts and miR-6835.

## Supporting information

S1 File Editing service certification. Our manuscript was edited for English language usage, grammar, spelling and punctuation by one or more native English-speaking editors at Nature Research Editing Service. The Key is BE6A-3B8F-0AB7-BFF5-2DF7.
(PDF)
